# Mediation effect of insomnia symptoms between positive psychotic like experiences and suicidal ideation among Lebanese young adults

**DOI:** 10.1186/s12888-023-04778-w

**Published:** 2023-04-20

**Authors:** Nour Farah, Sahar Obeid, Diana Malaeb, Chadia Haddad, Feten Fekih-Romdhane, Souheil Hallit

**Affiliations:** 1 Faculty of Science, Lebanese University, Fanar, Lebanon; 2 School of Arts and Sciences, Social and Education Sciences Department, Lebanese American University, Jbeil, Lebanon; 3 College of Pharmacy, Gulf Medical University, Ajman, United Arab Emirates; 4 Research Department, Psychiatric Hospital of the Cross, Jal Eddib, Lebanon; 5 School of Health Sciences, Modern University for Business and Science, Beirut, Lebanon; 6 INSPECT-LB (Institut National de Santé Publique, d’Épidémiologie Clinique Et de Toxicologie-Liban), Beirut, Lebanon; 7 The Tunisian Center of Early Intervention in Psychosis, Department of Psychiatry “Ibn Omrane”, Razi Hospital, 2010 Manouba, Tunisia; 8 Faculty of Medicine of Tunis, Tunis El Manar University, Tunis, Tunisia; 9 School of Medicine and Medical Sciences, Holy Spirit University of Kaslik, P.O. Box 446, Jounieh, Lebanon; 10 Applied Science Research Center, Applied Science Private University, Amman, Jordan

**Keywords:** Lebanon, Psychotic-like experiences, Suicide, Insomnia

## Abstract

**Background:**

Psychotic symptoms reported by healthy individuals in the general population are referred to as psychotic-like experiences (PLEs) and have been proven to increase the risk of suicidal ideation (SI) in these individuals. As it is well established that PLEs and insomnia share a bidirectional association and also that insomnia is linked to SI, we hypothesized that insomnia may represent a mediator underlying the relationship between PLEs and SI. Our aim was to validate this hypothesis among Lebanese young adults.

**Methods:**

A total of 3103 young adults (mean age 21.73 ± 3.80 years; 63.6% females) recruited from all Lebanese governorates completed a self-administered online questionnaire. PLEs were assessed using the CAPE-42 scale, SI using the Columbia Suicide Rating Scale, and insomnia using the Insomnia Severity Index). We conducted a mediation analysis using SPSS PROCESS v3.4 model 4 with three pathways. Variables that showed a *p* < 0.25 in the bivariate analysis were entered in the path analysis.

**Results:**

A total of 1378 participants (44.4%) had insomnia; 18.8% had SI; 42.5% reported at least one positive PE ‘nearly always’, and 30.5% reported at least one negative PE with this frequency. The results of the mediation analysis showed that insomnia severity partially mediated the association between positive dimension and SI; higher positive dimension was significantly associated with more insomnia severity, which was, in turn, significantly associated with more SI. Finally, more positive dimension was significantly and directly associated with more SI.

**Conclusion:**

These preliminary findings might encourage the implementation of new preventive measures to reduce SI among PLEs patients. Treating symptoms of insomnia might help reduce the risk of suicide.

## Background

Suicide is a major public health problem. Despite huge preventive efforts of researchers and stakeholders, suicide deaths remain frequent worldwide [1, 2]. According to the World Health Organization, 16 people per 100,000 die from suicide each year [3] An estimated 1.4% of global deaths were from suicide in 2017 [1]. In addition, suicide is consistently found to be the second leading cause of death among young adults (aged between 15 and 29 years) globally [4–6]. Among them, the vast majority (78%) occur in low- and middle-income countries [7]. Previous studies have particularly shown high prevalence rates of suicidal ideation (SI) among adolescents and young adults in Lebanon [8, 9]. The majority of suicides are related to mental health problems, with psychosis being among the most relevant risk factors [10]. In particular, increasing evidence from longitudinal studies evidenced a significant and positive relationship between PLEs and suicidal ideation and behavior (SIB) [11–14].

Psychotic-like experiences (PLEs) can be defined as subthreshold psychotic symptoms (delusions and hallucinations) that may be distressing but do not motivate help-seeking [15]. PLEs are at the mildest end of the psychosis continuum (which ranges from subclinical psychotic symptoms in the general population to more severe psychotic disorders); and are associated with a heightened risk of developing later psychosis [16]. A meta-analysis of epidemiological evidence on psychotic experiences in children and adults [17] revealed a median annual incidence of 2.5% and a prevalence of 7.2%. In a cross-national analysis of PLEs [18] which assessed prevalence and correlates of PLEs across 18 participating countries (including Lebanon and Iraq in the Middle East) a mean lifetime prevalence of ever having a lifetime PLEs was 5.8%. Another study in Qatar [19] showed that the prevalence of PLEs was 27.9%. Beyond psychotic disorders, PLEs have been shown to lead to an increased risk of other mental disorders [20], such as depression and anxiety disorders [21], substance use disorders, and suicidal behavior [22]. Indeed, it has been demonstrated that suicidal ideation (SI) and behaviors are commonly reported by young individuals from the general population who also self-report PLEs [23, 24]. However, while studies demonstrating the association of PLEs with clinically diagnosed psychosis have been extensive, there is few amount of research on the mechanisms underlying the relation between PLEs and non-psychotic psychopathologies, including SIB.

### The relationship between PLEs and SI

SI refers to thoughts and cognition ranging from vague ideas that life is not worth living, to a specific plan to commit suicide. A growing amount of research documented the causal association between PLEs and subsequent SI. For instance, a cohort study conducted in Sweden on young adolescents [25] revealed that PLEs co-occurrence predicted a sixfold increased risk of persistence of SI. Another research assessing psychotic symptoms as a clinical marker of risk for suicide attempt demonstrated 70-fold increased risk of suicide attempts in adolescents with psychopathology who reported PLEs [12]. Bromet et al. [14] investigated the possible influence of mental disorders on the associations between PLEs and SIB and it was revealed that PLEs increase the risk of SIB independently of antecedent mental disorders. Even while statistical adjustments removed the association between PLEs and suicidal thoughts, PLEs incidence did significantly increase the probability of suicide attempts when common risk factors were taken into account [26]. Overall, a recent meta-analysis encompassing ten prospective cohort studies published since 2013 concluded that subjects who experienced PLEs showed 2-, 3- and fourfold increases in subsequent SI, suicide attempt, and suicide death, respectively [11]. Interestingly, research found that PLEs significantly and consistently predict later suicidal behavior among people with SI [27], which highlights their high clinical relevance as markers of suicide risk. Hence, the importance of deepening our knowledge on the factors that play a mediating role in the interplay between PLEs and SI. Furthermore, specific PLEs seem to be differentially associated with SI. For example, perceptual anomalies and bizarre experiences have been found to relate more than other PLEs domains to SI [28]. Other previous findings revealed that positive psychotic symptoms were linked to increased risks of SI and suicide attempt, whereas negative psychotic symptoms were related to reduced risks of these outcomes [29]. However, the association between negative symptoms and suicidality is not well-established; with either positive [30], negative [31, 32] or no significant [33, 34] relationships found between them.

Potential shared underlying risk factors to co-existing PLEs and suicidality have been discussed, including shared genetic predispositions or environmental risk factors (e.g., childhood trauma) [27] However, some previous studies found that confounding effects of trauma and victimization did not explain the link between PLEs and suicidality [35, 36] Other putative mechanisms may also be involved, such as emotional reactivity to stress [37], traumatic brain injury [38], as well as psychological distress related to PLEs themselves [39]. Another possible mechanism include nightmares, which were involved in increased risks for both PLEs and SI [40–43]. In this regard, a range of psychological and psychopathological mediators have been previously investigated, including mental disorders, coping skills, affective reactions, mood stability and self-esteem [11]. However, no previous studies have explored the mediating effect of insomnia, to the best of our knowledge. We thus intend to add to the body of knowledge by examining insomnia as a theoretically based mediator in the relationship between PLEs and SI.

### Insomnia as mediator between PLES and SI

Insomnia can be defined as “a repeated difficulty with sleep initiation, duration, consolidation, or quality that occurs despite adequate time and opportunity for sleep and results in some form of daytime impairment and lasting for at least one month” [44]. Insomnia represents one of the most common sleeping disorders worldwide [45], with a prevalence in the general population of 10–25% [46–48].Based on literature data, we propose the hypothesis that insomnia may represent a mediator underlying the relationship between PLEs and SI. Insomnia is associated with both PLEs [49–51] and SIB [52, 53]. As for SIB, it is now well-established that insomnia is linked to SI [54–56], suicide attempts [57, 58], and even suicide deaths [59, 60] in adolescents and young adults. Recent research has also demonstrated that insomnia symptoms independently predict the risk for SIB after adjusting for drug/alcohol dependence and mental disorders [52, 58, 61]. The current literature mainly describes the relationship between insomnia and suicide in many psychopathologies but also in patients suffering from non-affective psychosis. The findings of a study conducted by Miller et al. [62] confirm once more that insomnia is robustly associated with suicide but it also extends this association onto patients affected by non-affective psychosis. With regard to PLEs, previous studies have shown that PLEs are significant predictors of insomnia symptoms and correlate as well with its severity [63]. This relationship between insomnia and PLEs has been demonstrated along the psychosis continuum, from clinical populations [64] to healthy individuals [65]. A longitudinal study has, for example, shown that insomnia was a strong predictor of paranoia, and helped predict to some extent hallucinations over a number of months in a clinical population [64]. Göder et al. [66] demonstrated in that there were significantly higher scores of magical and delusional ideations in patients with insomnia compared to healthy controls.

### The present study

As of late, Lebanese young adults have been heavily exposed to several risk factors of both PLEs and suicide because of the many crises the country has been going through. The alarming mental health status in Lebanon has been referred to as “Tomorrow's silent epidemic” [67]. Studies have for example shown that 28.9% of Lebanese adolescents reported some type of SI [9]. Another study found that 78.4% of university students had a clinically significant insomnia [68]. No studies have investigated to date PLEs in a Lebanese population to our knowledge. Furthermore, despite the consistent available data on the significant paths linking PLEs to insomnia and insomnia to SI, no previous study has tested the possibility of a mediating effect of insomnia symptoms in the association between PLEs and SI. As such, we believe that this study contributes to the existing literature in different ways. Our objectives were to test the hypothesis that insomnia severity mediates the positive association between PLEs and SI in a large sample of non-clinical Lebanese young adults from the general population.

## Methods

### Participants

This cross-sectional study was carried out between June and August 2022. A total of 3103 young adults was recruited from all Lebanese governorates. The research team contacted the administration of several universities to disseminate the link to all students registered. Participants received an online link to the survey. The link contained the consent form, information form (purpose of the current study, anonymity, voluntariness of consent to research), and the questionnaire. All participants responded willingly to the survey. There were no fees for participating in the study. Participants were included if they: (1) were aged between 18–35 years (because the ultrahigh-risk for psychosis population predominantly belongs to this age range [69], (2) had no self-reported physician-diagnosis of mental illness, including psychosis, and (3) had no previous antipsychotic intake. Excluded were those who refused to complete the survey. In addition, to address the goal of the present study, we excluded individuals with a self-reported physician’s mental illness diagnosis, and this, for two reasons. According to the continuum of psychosis, psychotic symptoms exist on a continuum across non-clinical individuals from the general population [70]. In addition, mental illnesses (such as depression or anxiety) may interfere with both insomnia [71] and suicidal ideation [72].

### Assessment

The questionnaire’s first section was about socio-demographic and other characteristics: age, gender, presence of a diagnosed psychiatric disorder, cigarette and alcohol usage, and lifetime drug use.

The second section included three measures: The Community Assessment of Psychic Experiences scale (CAPE-42), the Columbia Suicide Rating Scale (C-SSRS), and the Insomnia Severity Index (ISI).

#### The CAPE-42

This is a 42-item self-report questionnaire measuring positive and negative psychotic symptoms and depressive symptoms on a two dimensional scale. The first dimension measures the frequency of symptoms on a four-point scale of ‘never’ = 1, ‘sometimes’ = 2, ‘often’ = 3 and ‘nearly always’ = 4, and the second dimension measures the degree of distress caused by the experience: ‘not distressed’ = 1, ‘a bit distressed’ = 2, ‘quite distressed’ = 3 and ‘very distressed’ = 4. The total score ranges from 42 to 168 on both dimensions. The positive subscale counts 20 items (range 20 -80 on both dimensions), the negative subscale 14 items (range 14 – 56 on both dimensions) and the depressive subscale 8 items (range 8 – 32 on both dimensions). In this study, only the positive dimension was used (McDonald’s omega = 0.81). To evaluate the prevalence of PEs, answers were recoded to 0 (never) and 1 (at least sometimes). The Arabic validated version of the CAPE-42 has been used in this study [73].

#### The C-SSRS

This scale was designed by investigators in the United States to distinguish the spheres of SIB. The scale is composed of 5 questions rated as yes/no; scores range from 0 to 5, with a score indicating no SI and higher scores indicating higher SI. This scale is validated in Arabic among adults [8] and adolescents [9] (McDonald’s omega = 0.79).

#### The ISI

This is a self-report questionnaire comprising of 7 items: severity of sleep onset, sleep maintenance, and early morning awakening problems, sleep dissatisfaction, interference of sleep difficulties with daytime functioning, noticeability of sleep problems by others, and distress caused by the sleep difficulties. ISI evaluates the nature, severity and impact of insomnia. It has been previously evaluated as a reliable scale for assessing the psychometric properties of insomnia [74]. Total scores vary from 0 to 28; a cutoff score of 10 (ISI ≥ 10) is indicative of insomnia cases in community individuals [75]. This scale has also been previously validated in Arabic in Lebanon [76] (McDonald’s omega = 0.85).

### Statistical analysis

SPSS software version 23 was used to conduct data analysis. We had no missing data in our database. McDonald’s omega values were recorded for reliability analysis of all scales and subscales. The Student t test was used to compare two means, whereas the Chi-square test used to compare two categorical variables. To check for a significant indirect effect of insomnia severity between PLEs and SI, we conducted a mediation analysis using SPSS PROCESS v3.4 model 4 with three pathways; pathway A from the independent variable to the mediator, pathway B from the mediator to the dependent variable and pathway C from the independent to the dependent variable. Variables that showed a *p* < 0.25 in the bivariate analysis were entered in the path analysis. Significance was set at a *p* < 0.05.

## Results

A total of 4158 participants filled the survey; 1055 were excluded for having self-reported mental health issues; the data of 3103 participants was analyzed consequently. The mean age of the sample was 21.73 ± 3.80 years (min = 18; max = 35), with 63.6% females. All sociodemographic and other characteristics of the participants are summarized in Table 1. A total of 1378 participant (44.4%) had insomnia. To evaluate the prevalence of PEs, answers were recoded to 0 (never) and 1 (at least sometimes). Among the positive items, those assessing Persecutory Ideation (PI) had the highest prevalence (96.6%), followed by items measuring magical thinking (92.3%). Bizarre Experiences (BE) and Perceptual Abnormalities (PA) were reported at least sometimes by 84.1% and 27.9% of participants, respectively. When the frequency increased to “nearly always, the prevalence of these positive dimensions decreased sharply (BE = 11.9%, PI = 21.7%, MT = 26.3%, PA = 1.3%). In addition, 42.5% of participants reported at least one positive PE ‘nearly always’, and 30.5% reported at least one negative PE with this frequency. Finally, 584 (18.8%) of the participants had SI.

**Table 1:** Sociodemographic and other characteristics of the participants (*N* = 3103)

Variable	N (%)
Gender	
Male	1130 (36.4%)
Female	1973 (63.6%)
Marital status	
Single	2800 (90.2%)
Married	303 (9.8%)
Education	
Secondary or less	159 (5.1%)
University	2944 (94.9%)
Housing area	
Urban	1498 (48.3%)
Rural	1605 (51.7%)
Living situation	
Alone	117 (3.8%)
With family	2962 (95.5%)
With friends	24 (0.8%)
Electronic cigarettes smoking	
No	2879 (92.8%)
Yes	224 (7.2%)
Cigarettes smoking	
No	2749 (88.6%)
Yes	354 (11.4%)
Waterpipe smoking	
No	2267 (73.1%)
Yes	836 (26.9%)
Alcohol drinking	
No	2645 (85.2%)
Yes	458 (14.8%)
Marijuana use	
No	3066 (98.8%)
Yes	37 (1.2%)
Other illegal drug use	
No	3083 (99.4%)
Yes	20 (0.6%)
	**Mean ± SD**
Age (in years)	21.73 ± 3.80
Household crowding index (person/room)	1.51 ± 0.72
Financial burden	5.98 ± 2.64
Insomnia severity index	9.07 ± 6.04
Smartphone addiction	29.32 ± 12.04
Suicidal ideation	0.26 ± 0.67
Positive CAPE dimension	31.59 ± 6.27

*CAPE* Community Assessment of Psychic Experiences

### Bivariate analysis

The results of the bivariate analysis are summarized in Tables 2 and 3. Higher SI was found in females compared to males and in single participants compared to married ones. Furthermore, higher positive dimension scores, insomnia severity, financial burden and household crowding index were significantly associated with more SI.

**Table 2:** Bivariate analysis of factors associated with suicidal ideation (*N* = 3103)

Variable	Mean ± SD	*p*	t	*df*
Gender		**0.006**	2.730	2442.83
Male	0.22 ± 0.65			
Female	0.28 ± 0.69			
Marital status		**0.005**	2.824	457.19
Single	0.27 ± 0.69			
Married	0.18 ± 0.47			
Education		0.996	0.005	3101
Secondary or less	0.26 ± 0.73			
University	0.26 ± 0.67			
Housing area		0.330	0.975	3101
Urban	0.27 ± 0.69			
Rural	0.25 ± 0.66			
Living situation		0.101	2.298	3100
Alone	0.38 ± 0.93			
With family	0.25 ± 0.66			
With friends	0.13 ± 0.34			
Electronic cigarettes smoking		0.985	0.018	3101
No	0.26 ± 0.66			
Yes	0.26 ± 0.81			
Cigarettes smoking		0.524	0.638	3101
No	0.26 ± 0.66			
Yes	0.28 ± 0.75			
Waterpipe smoking		0.991	0.012	3101
No	0.26 ± 0.68			
Yes	0.26 ± 0.65			
Alcohol drinking		0.224	1.217	3101
No	0.26 ± 0.68			
Yes	0.22 ± 0.65			
Marijuana use		0.548	0.600	3101
No	0.26 ± 0.67			
Yes	0.32 ± 0.91			
Other illegal drug use		0.152	1.491	19.079
No	0.26 ± 0.67			
Yes	0.65 ± 1.18			

Numbers in bold indicate significant *p* values

**Table 3:** Correlation of continuous variables with suicidal ideation

	**1**	**2**	**3**	**4**	**5**	**6**	**7**
1. Suicidal ideation	1						
2. Positive dimension	.26***	1					
3. Age	-.02	-.01	1				
4. Household crowding index	.05**	.07***	-.17***	1			
5. Financial burden	.04*	.08***	.01	.11***	1		
6. Insomnia severity index	.18***	.36***	.03	.03	.03	1	
7. Negative dimension	.24***	.55***	-.01	-.01	.07***	.41***	1

^**^p < .01

^***^p < .001; numbers in the table refer to Pearson correlation coefficients

### Mediation analysis

**Fig. 1:**
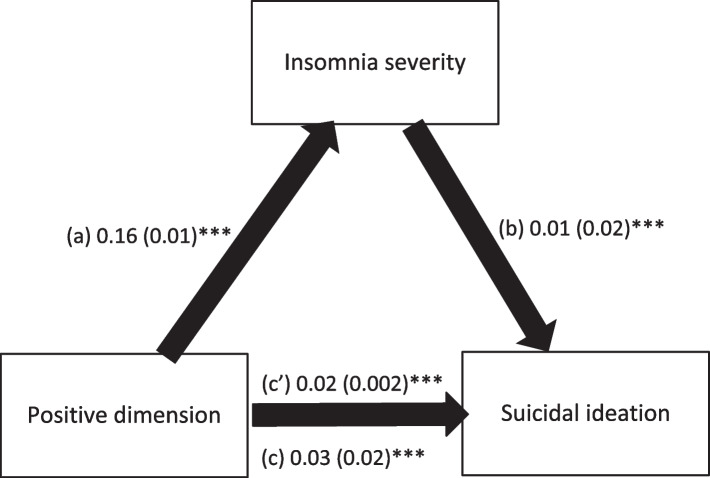
**a** Relation between positive dimension and insomnia severity (R^2^ = .361%); (**b**) Relation between insomnia severity and suicidal ideation (R^2^ = .084%); (**c**) Total effect of positive dimension on suicidal ideation (R^2^ = .075%); (c’) Direct effect of positive dimension on suicidal ideation. Numbers are displayed as regression coefficients (standard error). ***p < 0.001

The mediation analysis was adjusted over the following variables: gender, marital status, living situation, alcohol drinking, other illegal drug use, household crowding index, and financial burden. The results of the mediation analysis showed that insomnia severity partially mediated the association between positive dimension and SI (Table 4). Higher positive dimension was significantly associated with more insomnia severity, which was, in turn, significantly associated with more SI. Finally, more positive dimension was significantly and directly associated with more SI (Fig. 1).

**Table 4:** Mediation analyses results, taking the positive dimension score as the independent variable, insomnia severity as the mediator and suicidal ideation as the dependent variable

	**Direct effect**			**Indirect effect**		
	**Beta**	**SE**	***P***	**Beta**	**Boot SE**	**Boot CI**
Insomnia	.02	.002	< .001	.004	.001	.002; .006*

^*^indicates significant mediation. Direct effect refers to the direct association between PLEs and suicidal ideation, whereas the indirect effect refers to the association between PLEs and suicidal ideation through insomnia severity

## Discussion

Regarding the direct effect, our results demonstrated that greater positive psychotic symptoms were directly associated with higher SI scores. This correlates with previous studies that also confirmed the association between PLES and suicidal thoughts and behavior [77]. Saha et al. demonstrated that the correlation between delusional like experiences and SI remained even after controlling other psychological disorders [36]. While the causality of the association between PLEs and SI remains unclear, several studies robustly confirm that PLEs is a risk factor of SI with and without other cofounding factors [11, 12, 78].

As for the indirect effect, our mediation analysis confirmed the hypothesis posed: insomnia symptoms are mediating variables of the association between PLEs and SI. A more severe insomnia in individuals having positive symptoms of PLEs lead to a more SI. This correlates with previous findings [62, 66, 79]. Moreover, it was also demonstrated that more SI was significantly associated with higher insomnia severity and directly associated to PLEs positive dimension scores. These outcomes are in agreement with previous studies that discussed the association of SI with both insomnia [72, 80, 81] and PLES [78, 82]. The mediation analysis conducted in this study linked these three variables (PLEs, SI and insomnia) and demonstrated the mediator role of insomnia in this association which extends the findings of previous literature. Consistent with our findings, a recent investigation revealed a moderator role of a subjective sleep parameter, i.e. sleep quality, in the association between SI and PLEs among university students [83]. However, sleep quality is a subjective perception and is different from insomnia, since the latter is rather a disorder that implies a diagnosis. Besides, among all sleep problems, insomnia symptoms are the strongest predictors of SI [84]. A broad range of theoretical mechanisms have been proposed to explain the link between insomnia and suicidality (for review, see [80]). Explaining mechanisms include biological and physiological factors (i.e., abnormalities in serotonergic function, hypothalamic–pituitary–adrenal axis dysregulation), chronotype and nightmares [80]. Psychological mechanisms have also been hypothesized, including hopelessness and dysfunctional beliefs about Sleep [80]. According to Harvey’s cognitive model of insomnia, individuals with insomnia tend to negatively tone cognitive activity; which triggers, in turn, emotional distress [85]. It is of note that insomnia mediated only partially the relationship PLEs-SI. This may be explained by the fact that other potential mediators or confounders seem to be involved in this association (e.g., mental disorders, mood stability, affective reactions, coping skills, and self-esteem [11]).

By specifically examining insomnia, we thus confirm and extend these earlier findings. These results preliminarily open up new opportunities in the reduction of SI in individuals experiencing positive PLEs. Nevertheless, we emphasize that our data is cross-sectional. As such, the present estimations of a mediation effect are rather correlational in nature, and the correct causal ordering assumption cannot be tested. It is of note that mediation analysis may be conducted if the temporal ordering of the variables is well-known [86, 87]. Strong evidence from several prospective studies supported the causal positive relationship leading from PLEs to suicidality [12–14, 26]; and from insomnia to suicidality [52, 53, 55, 59–61]. We are aware that our findings are only preliminary, and we caution readers against interpreting these results causally, until future longitudinal research confirms our findings.

### Clinical implications

This current study has revealed the role of mediator that insomnia plays in the relationship between PLEs and SI. This would imply that practitioners working with people at risk of suicide attempts would benefit from screening not only for PLEs symptoms but also for insomnia. Treating insomnia in individuals exhibiting positive PLEs may aid in lowering SI in this population. While controlling PLES symptoms might be more challenging, treating insomnia and is a more attainable goal as insomnia is a modifiable factor with established effective non-pharmacological treatments. Indeed, many studies have confirmed that CBT for treating insomnia has lowered both SI [88, 89] and positive dimension symptoms in PLES patients [90]. Other evidence showed that managing insomnia through controlled-release zolpidem [91] can improve SI.

### Limitations and perspectives for future research

Some limitations of this study have to be mentioned. First, the snowball sampling technique used in this study might lead to a selection bias. In addition, the percentage of non-response rate is unknown. As this article follows the cross-sectional design, this prevents us from establishing causality between the variables. An information bias might also be present since participants tend to falsify the information given during surveys. A residual confounding bias is possible since not all factors associated with PLEs were considered for this study. Additionally, we limited the number of items of the questionnaire to those that were relevant to our objectives, in order to reduce the administration time and burden. We thus did not take other already known mediators into account (e.g., depressive and anxiety symptoms, nightmares). While limiting the items appeared to contribute to reaching a large sample size, future studies could examine other possible psychiatric symptoms associated with both PLEs and SI. Moreover, future studies should consider controlling for co-occurring sleep disorders. The sample is mostly composed of females. Also, the effects of the COVID-19 pandemic may have heightened symptoms in general. Another limitation lies to the fact that positive PLEs was considered as a unique dimension in this study, whereas specific PLEs domains could be differentially linked to SI [28]. Additional research should consider using statistical approaches such as network analysis or latent profile analysis to better understand the specific associations between each PLEs’ nature/severity and SI. Finally, although SI is a strong predictor of subsequent suicide behaviors [92, 93], future studies are needed to investigate the interplay between PLEs, insomnia and suicidal behaviors (e.g., past suicide attempts or suicide plans).

## Conclusion

As a partial mediator role of insomnia in the cross-sectional association between PLEs and SI was preliminarily confirmed in this study, clinical practitioners would benefit from preventing death by suicide by screening for and treating insomnia symptoms in individuals who self-report both PLEs and SI. Future studies establishing the causality between the variables would be valuable in complementing this research.

## Data Availability

All data generated or analyzed during this study are not publicly available due the restrictions from the ethics committee. Reasonable requests can be addressed to the corresponding author.
